# *Quercus acuta* Thunb. Suppresses LPS-Induced Neuroinflammation in BV2 Microglial Cells via Regulating MAPK/NF-κB and Nrf2/HO-1 Pathway

**DOI:** 10.3390/antiox11101851

**Published:** 2022-09-20

**Authors:** Jae Kwang Kim, Hye Jin Yang, Younghoon Go

**Affiliations:** Korean Medicine (KM)-Application Center, Korea Institute of Oriental Medicine (KIOM), Daegu 41062, Korea

**Keywords:** *Quercus acuta* Thunb., neuroinflammation, microglia, NF-κB, MAPK, Nrf2, HO-1

## Abstract

Microglial activation-mediated neuroinflammation is associated with the pathogenesis of neurodegenerative disorders. Therefore, the management of microglial cell activation and their inflammatory response is an important therapeutic approach for preventing neurodegenerative diseases. *Quercus acuta* Thunb. (QA) (*Fagaceae*) is a tree found in Korea, China, and Japan. The current study investigated the anti-neuroinflammatory effects of QA and its mechanism of action in lipopolysaccharide (LPS)-stimulated BV2 microglial cells. Pretreatment with a methanol extract of dried QA stems (QAE) inhibited the production of nitric oxide and proinflammatory cytokines and decreased the expression of inducible nitric oxide synthase, cyclooxygenase-2 in LPS-stimulated BV2 microglial cells. Furthermore, it inhibited the phosphorylation and degradation of inhibitory κBα and decreased the nuclear translocation and phosphorylation of nuclear factor-κB (NF-κB). Moreover, QAE inhibited the phosphorylation of extracellular signal-regulated kinase, p38 and c-Jun N-terminal kinase, which is known as mitogen-activated protein kinase (MAPK). Additionally, QAE treatment increased heme oxygenase-1 (HO-1) expression by activating the nuclear factor erythroid 2-related factor 2 (Nrf2) signaling, thereby ameliorating LPS-induced intracellular hydrogen peroxide production. Finally, it was found that catechin and taxifolin, two phytochemicals of QAE, also reduced the expression of inflammatory mediators. These findings suggest that QA is beneficial for preventing microglia-mediated neuroinflammatory response through the inhibition of NF-κB, MAPK and the activation of Nrf2/HO-1 signaling pathways.

## 1. Introduction

Microglia, innate immune effector cells that reside in the central nerve system (CNS), perform pivotal roles in brain homeostasis. Microglia exert neuroprotective functions in the healthy brain by providing innate immunity, removing apoptotic cells and modifying synaptic connectivity [1]. On the other hand, stimuli such as pathogenic insults, traumatic brain injury and amyloid, tau protein activate the microglia, prompting them to release cytokines, chemokines and reactive oxygen species (ROS) [2]. Microglial activation-driven neuroinflammation eventually causes neuronal cell death and synaptic dysfunction since neurons are vulnerable to these inflammatory mediators [1,3]. Given that increased microglial activation is positively correlated with neuronal dysfunction and abnormal protein aggregation in patients with Alzheimer’s and Parkinson’s disease, regulating the inflammatory response of microglia is regarded as an important prophylactic target to prevent the development and progress of neurodegenerative disease [4,5].

Microglial cells mediate an inflammatory response against pathogenic stimuli by recognizing pathogen-associated molecular patterns or damage-associated molecular patterns via toll-like receptor (TLR) [6]. Endotoxin binding to TLR on the surface of microglia induces intracellular signaling transduction, which activates the nuclear factor-κB (NF-κB) signaling pathway [7]. NF-κB is a key transcription factor that regulates immune response, inflammation and cell survival. NF-κB signaling activation in microglial cells increases the transcription of proinflammatory enzymes such as inducible nitric oxide synthase (iNOS) and cyclooxygenase-2 (COX-2) and cytokines, eliciting neuroinflammation [2,8,9].

ROS plays a role in cell signaling transmission and homeostasis maintenance. As a secondary messenger, ROS contributes to the modulation of microglial phagocytic activity and inflammatory response [10]. Proinflammatory stimulation induces the interaction between TLR and NADPH oxidase (NOX), causing intracellular ROS generation [11,12]. The induction of intracellular H_2_O_2_ facilitates inflammatory response by activating NF-κB and MAPK pathways [10,12]. Numerous studies have found that inducing antioxidant proteins such as heme oxygenase-1 (HO-1) by activation of the nuclear factor erythroid-2-related factor 2 (Nrf2), ameliorates inflammatory response in microglia and macrophages [12,13,14]. Therefore, several medicinal plants and natural products with anti-inflammatory and antioxidative stress properties, have gained interest as therapeutic agents in inflammation-related disorders.

The tree *Quercus acuta* Thunb. (QA) is found in East Asia including Korea, China and Japan [15]. Many species of the genus *Quercus* have been used as medicinal plants to treat septic and gastrointestinal disorders [16]. Previous research on the biological efficacy of QA has shown that the leaf extract of QA has anti-hyperuricemia, antibacterial properties [15,17]. Furthermore, phenolic compounds isolated from QA stem such as catechin, epicatechin and taxifolin have exhibited anti-inflammatory and radical scavenging activity [18]. Moreover, the anti-viral effect of QA on herpes simplex virus-1 (HSV-1) replication via the inhibition of ROS generation and NF-κB signaling has been studied [19]. Despite the fact that QA has antioxidant and anti-inflammatory characteristics, its pharmacological action in activated microglia is unknown. Therefore, the present study investigated the anti-neuroinflammatory activity of QA in lipopolysaccharide (LPS)-stimulated BV2 microglial cells. Furthermore, we explored the regulatory effect of QA on NF-κB, MAPK and Nrf2/HO-1 signaling pathways to determine the molecular mechanisms involved.

## 2. Materials and Methods

### 2.1. Materials and Reagents

Methanol extract of dried QA stems (QAE) (Voucher No. PB2418) was provided by the Korea Plant Extract Bank (Cheongju, Republic of Korea), and dissolved in dimethyl sulfoxide (100 mg/mL) for cell treatment. Anti-iNOS (NB300-605) antibody was obtained from Novus Biologicals (Centennial, CO, USA). Anti-COX-2 (#12282), anti-p65 (#8242), anti-phosphorylated p65 (Ser^536^) (#3033), anti-inhibitory κBα (IκBα) (#4814), anti-phosphorylated IκBα (#9246), anti-TATA-box binding protein (TBP) (#8515), anti-extracellular signal-regulated kinase (ERK) (#4377), anti-phosphorylated ERK (Thr^202^/Tyr^204^) (#9102), anti-p38 (#9212), anti-phosphorylated p38 (Thr^180^/Tyr^182^) (#9211), anti-c-Jun N-terminal kinase (JNK) (#9252), anti-phosphorylated JNK (Thr^183^/Tyr^185^) (#9251), anti-HO-1 (#82206) antibodies were purchased from Cell Signaling Technology (Beverly, MA, USA). Anti-β-actin (sc-81178), anti-Nrf2 (sc-722) antibodies were obtained from Santa Cruz Biotechnology (Santa Cruz, CA, USA). Anti-phosphorylated Nrf2 (Ser^40^) (ab76026), anti-glutamate-cysteine ligase catalytic subunit (GCLC) (ab190685) antibodies were from Abcam (Cambridge, MA, USA). Anti-Sestrin-2 (SESN2) (10795-1-AP) antibody was provided by Proteintech (Chicago, IL, USA). LPS from *Escherichia coli* and the reference standards of catechin and taxifolin (purity over 97%) used in ultra-high performance liquid chromatography coupled to high-resolution Orbitrap mass spectrometry (UHPLC-UV-HRMS) analysis were obtained from Sigma-Aldrich (St. Louis, MO, USA). MS-grade products, water, acetonitrile, and formic acid used as mobile phase were purchased from Thermo Fisher Scientific (Rockford, IL, USA).

### 2.2. Cell Culture

BV2, immortalized murine microglial cells, were kindly donated from Professor Kyungho Suk (Kyungpook National University, Daegu, Korea), and were cultured in Dulbecco’s modified Eagle’s medium (HyClone Laboratories, Logan, UT, USA) with 10% fetal bovine serum, penicillin (100 U/mL) and streptomycin (100 μg/mL) (HyClone Laboratories), under humidified conditions with 5% CO_2_, at 37 °C temperature.

### 2.3. Cell Viability Assay

BV2 microglial cells were plated in 96-well plates (30,000 cells/well) to determine cell viability. After treatment, cells were further incubated with CCK-8 (Dojindo Laboratories, Kumamoto, Japan) for 2 h. Absorbance at 450 nm was detected using an automated plate reader (SpectraMax i3, Molecular Devices, Sunnyvale, CA, USA). Relative cell viability was calculated using the following equation:

$$\mathrm{Relative}\;\mathrm{cell}\;\mathrm{viability}\;\left(\%\right)=\left(\frac{\mathrm{Absorbance}\;\mathrm{of}\;\mathrm{the}\;\mathrm{treated}\;\mathrm{group}}{\mathrm{Absorbance}\;\mathrm{of}\;\mathrm{the}\;\mathrm{control}\;\mathrm{group}}\right)\times 100$$

### 2.4. Measurement of Nitric Oxide (NO) Production

To measure NO production, conditioned media was reacted with the same volume of Griess reagent for 10 min. Absorbance at 550 nm was measured using an automated plate reader (Molecular Devices).

### 2.5. Preparation of Whole Cell Lysates and Nuclear Fraction Extract, and Immunoblot Assay

Cells were lysed with radioimmunoprecipitation (RIPA) buffer containing protease and phosphatase inhibitor cocktail (Roche Molecular Biochemicals, Mannheim, Germany), and incubated on ice for 40 min with vigorous vortex every 10 min. The cell lysate was centrifuged at 15,000× *g* for 30 min, and the supernatant was collected as whole cell lysates. Nuclear fraction was obtained using a commercial nuclear extraction kit (Thermo Fisher Scientific), according to the manufacturer’s instruction. The bicinchoninic acid assay was performed to determine the protein content (Thermo Fisher Scientific). An equal amount of protein was then separated using sodium dodecyl sulfate polyacrylamide gel electrophoresis (SDS-PAGE), and transferred to a polyvinylidene fluoride membrane (Millipore, Bedford, MA, USA). After blocking with 5% skim milk, the membrane was allowed to reacted with primary antibody overnight at 4 °C, and subsequently incubated with horseradish peroxide conjugated-secondary antibody (Cell Signaling Technology). The chemiluminescence density of the protein of interest was detected by the Alliance Q9 chemiluminescence imager (Uvitec, Cambridge, UK), and densitometric analysis was performed using ImageJ (US National Institutes of Health, Bethesda, MD, USA). Equal protein loading was verified by β-actin (whole cell lysates) or TBP (nuclear fraction) immunoblotting.

### 2.6. RNA Isolation and Real-Time PCR

Total RNA was extracted using QIAzol Lysis Reagent (Qiagen, Hilden, Germany), as directed by the manufacturer. Reverse transcription was conducted using Maxima Reverse Transcriptase (Thermo Fisher Scientific). Real-time PCR was performed using SYBR Green qPCR Master Mix (Thermo Fisher Scientific) and CFX real-time PCR detection system (Bio-Rad, Hercules, CA, USA). Glyceraldehyde-3-phophate dehydrogenase (*GAPDH*) was used as the reference gene. The primer sequences used for the RT-qPCR reaction are given in Table 1. Relative gene expression was calculated using the 2^−^^ΔΔ^^CT^ method [20].

### 2.7. Measurement of Cytokine Release

BV2 microglial cells were preincubated with QAE (25–100 μg/mL) for 3 h, and exposed to LPS for 12 h. After treatment, the level of cytokine in cultured media was measured using commercial enzyme-linked immunosorbent assay (ELISA) kit (Thermo Fisher Scientific) according to the manufacturer’s instruction.

### 2.8. Immunocytochemistry

Cells were seeded onto coverslips (Marienfeld, Lauda-Königshofen, Germany). After treatment, cells were fixed with 4% paraformaldehyde for 15 min, and permeabilized with 0.25% Triton X-100 for 30 min at room temperature. After blocking with 1% bovine serum albumin, cells were incubated with primary antibody (anti-p65) for overnight at 4 °C, and subsequently reacted with Alexa Fluor 488-conjugated secondary antibody (Thermo Fisher Scientific) at room temperature for 2 h. After staining nuclei with Hoechst 33342 (Thermo Fisher Scientific), coverslips were mounted with fluorescence mounting solution (Dako, Glostrup, Denmark), and observed under a Lionheart FX microscope (BioTek, Vermont, Winooski, VT, USA).

### 2.9. Measurement of H_2_O_2_ Production

After treatment, BV2 microglial cells were stained with H_2_DCF-DA (10 μM) for 30 min. The fluorescence intensity of dichlorofluorescein (DCF) was detected at a wavelength of 485/530 nm (emission/excitation) using an automated plate reader (Molecular Devices).

### 2.10. UHPLC-UV-HRMS Analysis

Phytochemical identification in QAE was performed by a Q-Exactive quadrupole Orbitrap mass spectrometer coupled with Thermo Dionex UltiMate 3000 system (UHPLC-UV-HRMS, Thermo Fisher Scientific). Chromatographic separation was carried out on a Waters Acquity BEH C18 column (2.1 mm i.d. × 100 mm, 1.7 µm) equipped with VanGuard XBridge BEH C18 pre-column (2.1 mm i.d. × 5 mm, 1.7 µm), with the oven temperature maintained at 40 °C. The mobile phase conditions for analysis were composed of 0.1% formic acid (*v/v*) in water (A) and acetonitrile (B), and the flow rate was maintained at 0.3 mL/min. Full MS and MS/MS spectrums were obtained via UHPLC-UV-HRMS that equipped with a heated electrospray ionization (HESI) interface. The data acquisition and analysis of all data obtained through this study were processed through Xcalibur v.4.2 and Tracefinder v.4.0 software (Thermo Fisher Scientific). In addition, the analysis method used in this study was referred and applied to the previously reported methods [21] and briefly described.

### 2.11. Statistical Analysis

To determine the significance difference among experimental groups, unpaired student’s *t*-test or one-way analysis of variance (ANOVA) was performed. According to results from one-way ANOVA, Tukey’s honest difference was conducted as post hoc analysis. All numerical data were expressed as bar chart (mean ± standard deviation) or box plot of at least three independent experiments. *p* Values under 0.05 were considered as statistically significant. The statistical analysis test was accomplished using GraphPad Prism 6.0 software (GraphPad Software, San Diego, CA, USA).

## 3. Results

### 3.1. QAE Inhibited NO Production in LPS-Stimulated BV2 Microglial Cells

**Figure 1:**
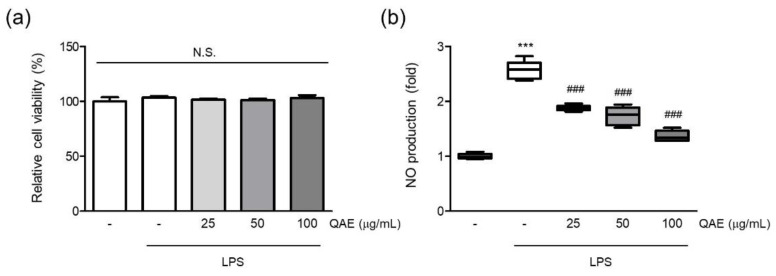
Methanol extract of *Quercus acuta* Thunb. stem (QAE) inhibits nitric oxide (NO) production in lipopolysaccharide (LPS)-stimulated BV2 microglial cells. BV2 microglial cells were pretreated with QAE (25–100 μg/mL) for 3 h, and then exposed to LPS (100 ng/mL) for 24 h. (**a**) Relative cell viability. After treatment, cell viability was measured using CCK-8 (*n* = 3). (**b**) NO production. Produced NO in media was determined by Griess assay (*n* = 5). Significant versus control, *** *p* < 0.001; Significant versus LPS alone-treated group, ^###^ *p* < 0.001; N.S., not significant.

First, the effect of QAE on cell viability was assessed to evaluate the cytotoxicity of QAE and the suitable dose on BV2 microglial cells. The final concentration of QAE up to 100 μg/mL (24 h) had no discernible cytotoxic effect on the viability of BV2 microglial cells (Figure 1a). Furthermore, the NO released in the medium was measured by Griess assay to determine whether QAE possessed any anti-inflammatory activity. Excessive NO production has been linked to the development of neurodegenerative diseases via nitrosative stress, protein misfolding, and neuronal damage [2,8]. After pretreatment with QAE (25–100 μg/mL, 3 h), BV2 microglial cells were exposed to LPS (100 ng/mL) for 24 h. As expected, the LPS treatment significantly increased NO production. Pretreatment with QAE (25–100 μg/mL) reduced NO generation in a dose-dependent manner (Figure 1b). Based on these results, further experiments were carried out at QAE concentrations in the range of 25–100 μg/mL.

### 3.2. QAE Suppressed iNOS and COX-2 Expression in LPS-Stimulated BV2 Microglial Cells

**Figure 2:**
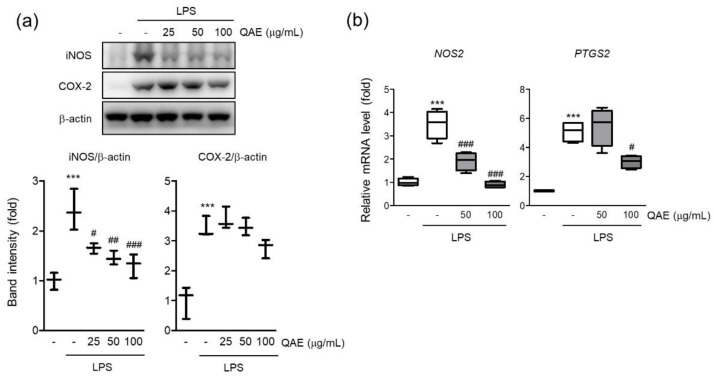
QAE decreases inducible nitric oxide synthase (iNOS) and cyclooxygenase-2 (COX-2) expression in LPS-stimulated BV2 microglial cells. BV2 microglial cells were pretreated with QAE (25–100 μg/mL) for 3 h, and further stimulated with LPS (100 ng/mL) for (**a**) 12 h, (**b**) 8 h. (**a**) Protein expression of iNOS and COX-2. β-actin immunoblotting was used to verify equal protein loading (*n* = 3). (**b**) mRNA expression of iNOS (*NOS2*) and COX-2 (*PTGS2*). Relative gene expression was quantified by RT-qPCR, and normalized by mouse *GAPDH* expression of each sample (*n* = 4). Significant versus control, *** *p* < 0.001; Significant versus LPS alone-treated group, ^#^ *p* < 0.05, ^##^ *p* < 0.01, ^###^ *p* < 0.001.

iNOS is a key enzyme responsible for inflammatory response by synthesizing NO from L-arginine [22]. The protein expression of iNOS was evaluated by immunoblot assay if the inhibitory effect of QAE on NO production was mediated by iNOS expression. As expected, LPS stimulation (100 ng/mL, 12 h) significantly increased the expression of iNOS (Figure 2a), and pretreatment with QAE (25–100 μg/mL) significantly suppressed iNOS protein level. According to protein expression, the mRNA level of iNOS was decreased by QAE pretreatment at 50 and 100 μg/mL (Figure 2b). COX-2 is an enzyme catalyzing the conversion of arachidonic acid to prostaglandins which act as an inflammatory mediator [9]. QAE (100 μg/mL) pretreatment decreased COX-2 protein expression (Figure 2a), and significantly suppressed mRNA expression of COX-2 (Figure 2b).

### 3.3. QAE Inhibited Proinflammatory Cytokine Production in LPS-Stimulated BV2 Microglial Cells

**Figure 3:**
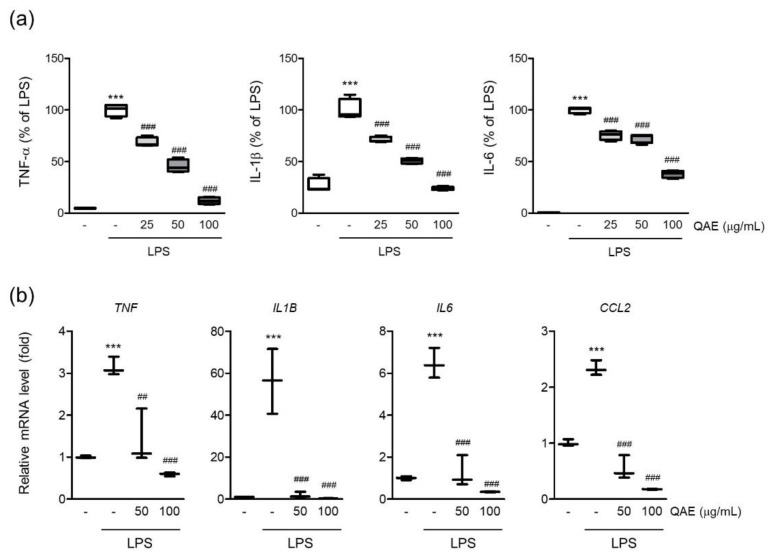
QAE inhibits proinflammatory cytokine expression in LPS-stimulated BV2 microglial cells. BV2 microglial cells were preincubated with QAE (25–100 μg/mL) for 3 h, and subsequently exposed to LPS (100 ng/mL) for (**a**) 12 h or (**b**) 8 h. (**a**) Level of proinflammatory cytokine production. The production of tumor necrosis factor-α (TNF-α), interleukin (IL)-1β, and IL-6 in conditioned media was determined by enzyme-linked immunosorbent assay (*n* = 4). (**b**) mRNA expression of proinflammatory cytokines. Relative mRNA expression of TNF-α (*TNF*), IL-1β (*IL1B*), IL-6 (*IL6*), and monocyte chemoattracted protein-1 (MCP-1) (*CCL2*) was measured by RT-qPCR, and normalized by *GAPDH* expression of each sample (*n* = 3). Significant versus control, *** *p* < 0.001; Significant versus LPS alone-treated group, ^##^ *p* < 0.01, ^###^ *p* < 0.001.

Microglia are the major source of proinflammatory cytokines in the brain. Endotoxin-driven TLRs pathway activation exacerbates neuroinflammation by expressing proinflammatory cytokines such as interleukin (IL)-1β, IL-6, and tumor necrosis factor-α (TNF-α) [6]. To determine the effect of QAE on the production of proinflammatory cytokines in LPS-stimulated BV2 microglial cells, the released levels of TNF-α, IL-1β, and IL-6 cytokines in conditioned media were measured by ELISA assay. As is well known, LPS stimulation (100 ng/mL for 12 h) significantly increases the expression of TNF-α, IL-1β and IL-6 in conditioned media. QAE pretreatment (25–100 μg/mL, 3 h) significantly decreased the expression of TNF-α, IL-1β, and IL-6 (Figure 3a). LPS stimulation (100 ng/mL, for 8 h) increased the mRNA levels of TNF-α, IL-1β, IL-6, and monocyte chemoattracted protein-1 (MCP-1), similar to cytokine release in conditioned media, whereas QAE pretreatment (50, 100 μg/mL, 3 h) significantly reduced cytokine mRNA expression (Figure 3b).

### 3.4. QAE Inhibited NF-κB Signaling Activation in LPS-Stimulated BV2 Microglial Cells

**Figure 4:**
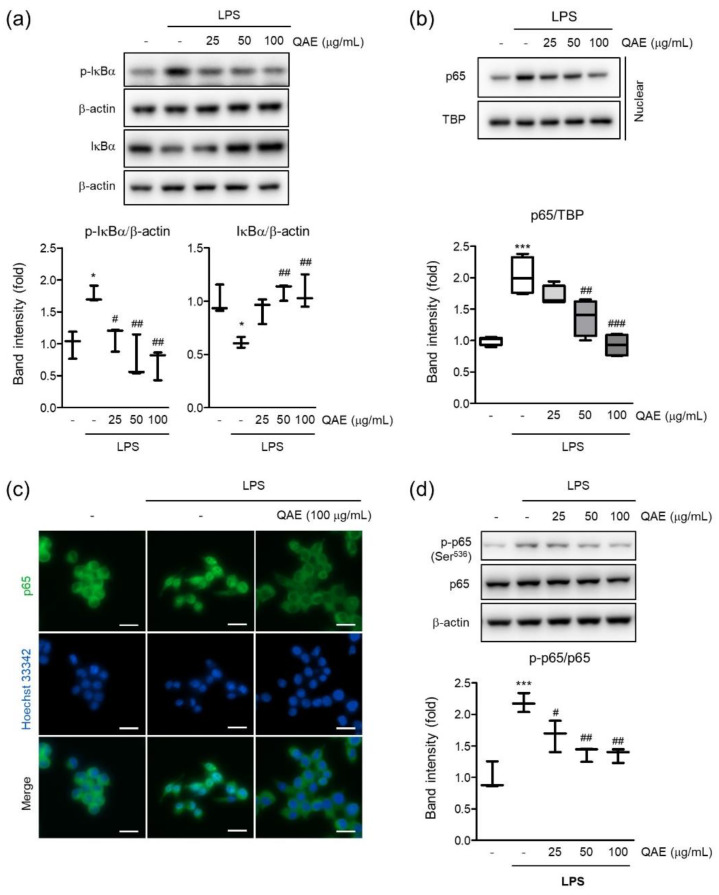
QAE inhibits the nuclear factor-κB (NF-κB) signaling pathway in LPS-stimulated BV2 microglial cells. After QAE pretreatment (25–100 μg/mL) for 3 h, BV2 microglial cells were exposed to LPS (100 ng/mL) for 1 h. (**a**) Phosphorylation and degradation of IκBα. Equal protein loading was verified by β-actin immunoblotting (*n* = 3). (**b**) p65 expression in nuclear. Equal nuclear protein loading was verified by TATA-box binding protein (TBP) immunoblot (*n* = 4). (**c**) Immunocytochemistry. After treatment, cells were immunostained with an anti-NF-κB (p65) antibody and Alexa Fluor 488-conjugated secondary antibody. Scale bar represents 25 μm. Hoechst 33342 was used for nuclear counterstaining (*n* = 3). (**d**) Phosphorylation of p65 at Ser^536^. Expression of phosphorylated p65 was normalized with expression of total p65 immunoblotting (*n* = 3). Significant versus control, * *p* < 0.05, *** *p* < 0.001; Significant versus LPS alone-treated group, ^#^ *p* < 0.05, ^##^ *p* < 0.01, ^###^ *p* < 0.001.

To determine the molecular mechanisms of the anti-inflammatory effect of QAE, we investigated the effect of QAE on the NF-κB signaling pathway in LPS-stimulated BV2 microglial cells. As expected, LPS treatment (100 ng/mL, 1 h) significantly increased phosphorylation and degradation of IκB. However, QAE pretreatment (50 and 100 μg/mL, 3 h) significantly inhibited IκB phosphorylation and inhibited degradation of IκB (Figure 4a). To investigate the effect of QAE on the nuclear translocation of NF-κB (p65 subunit), nuclear p65 was detected by immunoblotting and immunofluorescence staining using p65 subunit-specific antibody. LPS stimulation (100 ng/mL, 1 h) enhanced the nuclear translocation of p65, which was considerably inhibited by QAE pretreatment (50 or 100 μg/mL, 3 h) (Figure 4b,c). Furthermore, we examined the effect of QAE on phosphorylation at Ser^536^ of the p65 subunit by immunoblotting. It is reported that LPS-induced phosphorylation of the p65 subunit at Ser^536^ is essential to the elevation of NF-κB transcriptional activity [23]. QAE pretreatment (50, 100 μg/mL, 3 h) significantly inhibited phosphorylation at Ser^536^ of p65 (Figure 4d). Overall, these results suggest that QAE exhibited anti-inflammatory activity through NF-κB inhibition.

### 3.5. QAE Inhibited Activation of MAPK Signaling Pathway

**Figure 5:**
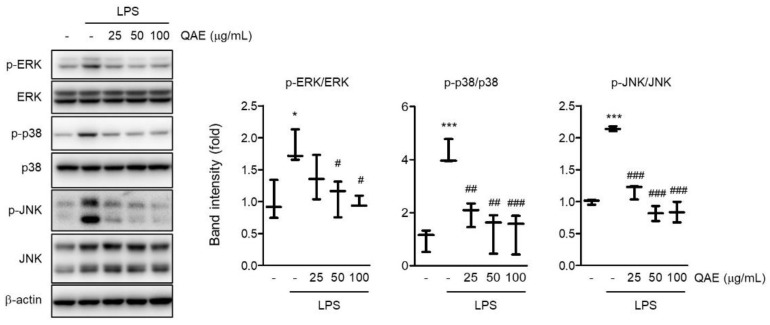
QAE inhibits the mitogen-activated protein kinase (MAPK) signaling pathway in LPS-stimulated BV2 microglial cells. After QAE pretreatment (25–100 μg/mL) for 3 h, BV2 microglial cells were exposed to LPS (100 ng/mL) for 1 h. Expressions of phosphorylated extracellular signal-regulated kinase (ERK), p38, and c-Jun N-terminal kinase (JNK) were normalized by expression of ERK, p38, and JNK protein expression, respectively (*n* = 3). Significant versus control, * *p* < 0.05, *** *p* < 0.001; Significant versus LPS alone-treated group, ^#^ *p* < 0.05, ^##^ *p* < 0.01, ^###^ *p* < 0.001.

MAPK signaling is well known to promote the production of inflammatory mediators [24]. To examine whether the anti-inflammatory activity of QAE is mediated by the abrogation of the MAPK signaling pathway, the phosphorylation of ERK, p38, and JNK was measured by immunoblot analysis. LPS stimulation (100 ng/mL, 1 h) significantly increased the phosphorylation of ERK, p38, and JNK. However, the phosphorylation of ERK, p38, and JNK was remarkably reduced in a dose-dependent manner by QAE pretreatment (25–100 μg/mL, 3 h) (Figure 5). These results suggest that QAE exhibited an inhibitory effect on the inflammatory response via inactivation of MAPK signaling.

### 3.6. QAE Decreased Cellular ROS via Nrf2/HO-1 Activation

**Figure 6:**
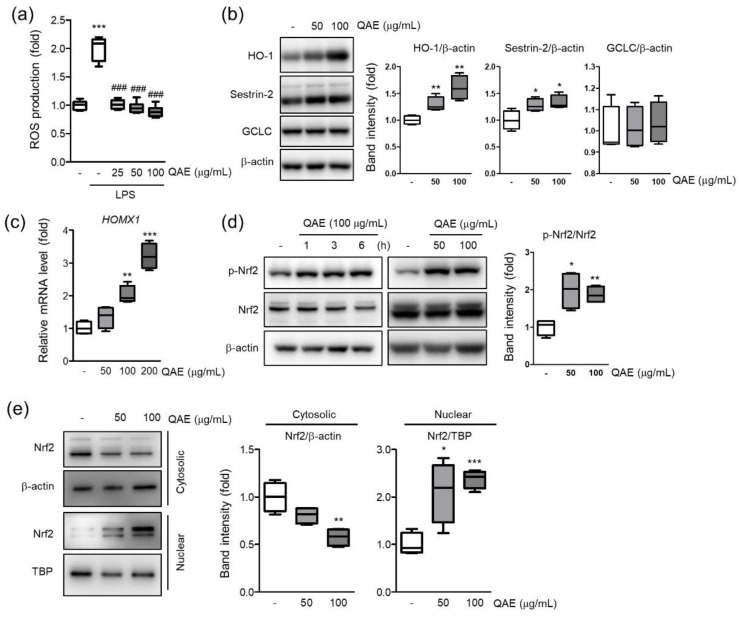
QAE activates the nuclear factor erythroid-2-related factor 2 (Nrf2)/heme oxygenase-1 (HO-1) signaling pathway in BV2 microglial cells. (**a**) H_2_O_2_ production. Cells were pretreated with QAE (25–100 μg/mL, 3 h), and further exposed to LPS (100 ng/mL, 12 h). After staining with H_2_DCF-DA (10 μM, 0.5 h), dichlorofluorescein (DCF) fluorescence intensity was detected using an automated plate reader (*n* = 8). (**b**) Expression of antioxidant protein. Cells were treated with QAE (50, 100 μg/mL) for 8 h. Equal loading of each protein was verified by β-actin immunoblotting (*n* = 4). (**c**) Heme oxygenase-1 (HO-1) (*HOMX1*) mRNA level. Cells were treated with QAE (50–200 μg/mL) for 7 h. Relative mRNA expression of HO-1 was normalized by expression of *GAPDH* (*n* = 4). (**d**) Phosphorylation of Nrf2. Cells were treated with 100 μg/mL of QAE for the indicated time (left), or 50, 100 μg/mL of QAE for 3 h (right) (*n* = 4). (**e**) Expression of Nrf2 in the cytoplasm and nuclear. Expressions of Nrf2 were normalized by β-actin (cytosolic) or TBP (nuclear) immunoblotting (*n* = 4). Significant versus control, * *p* < 0.05, ** *p* < 0.01, *** *p* < 0.001; Significant versus LPS alone-treated group, ^###^ *p* < 0.001.

Intracellular ROS accumulation by TLR signaling intensifies the inflammatory response [11]. To investigate whether QAE possess inhibitory activity on H_2_O_2_ production, intracellular H_2_O_2_ production was measured using H_2_DCF-DA in LPS (100 ng/mL, 12 h)-stimulated BV2 microglial cells. As expected, LPS treatment significantly increased the fluorescence intensity of DCF, which reflects the production of H_2_O_2_ in cells. However, QAE pretreatment (25–100 μg/mL, 3 h) significantly inhibited H_2_O_2_ production induced by LPS (Figure 6a). Further, the effect of QAE on antioxidant protein expressions such as HO-1, SESN2, and GCLC was examined. QAE treatment (50, 100 μg/mL, 8 h) significantly increased the protein expression of HO-1, SESN2 (Figure 6b). Accordingly, QAE treatment (100, 200 μg/mL, 7 h) significantly increased HO-1 mRNA level (Figure 6c). Because Nrf2 is widely recognized as a master transcriptional factor of antioxidant proteins genes such as HO-1, the phosphorylation of Nrf2 by QAE treatment was monitored. Results showed that the expression of total Nrf2 was constant, whereas the expression of phosphorylated Nrf2 was increased by QAE treatment (50, 100 μg/mL, 3 h) (Figure 6d). In addition, immunoblot analysis using cytosolic and nuclear fraction protein revealed that QAE treatment (50 and 100 μg/mL, 3 h) induced translocation of Nrf2 to the nucleus (Figure 6a). These results suggested that Nrf2 activation by QAE treatment elevates HO-1 expression and decreases H_2_O_2_ production.

### 3.7. Phytochemicals of QAE Inhibited Expression of Proinflammatory Mediators

**Figure 7:**
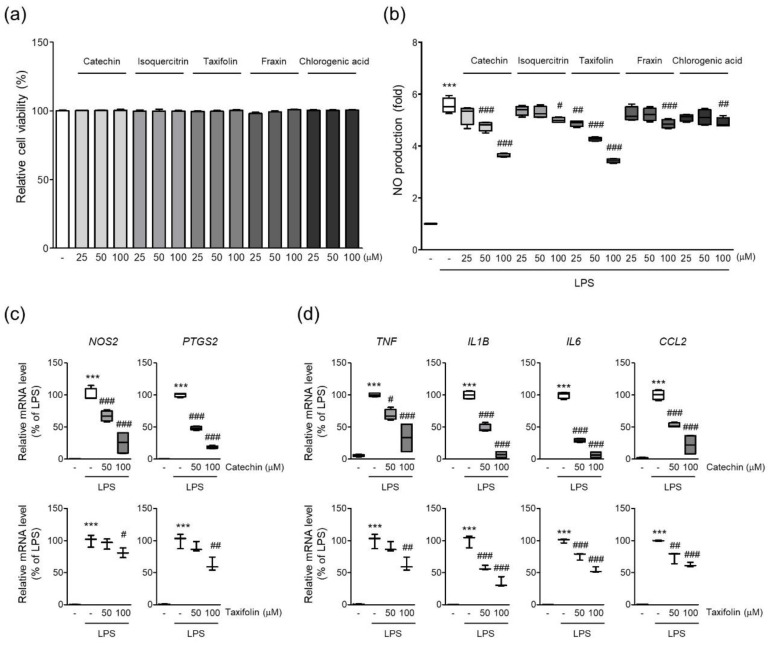
Phytochemicals of QAE inhibited the expression of proinflammatory mediators. (**a**) Relative cell viability. BV2 microglial cells were treated with each phytochemical at indicated dose for 24 h. Cell viability was determined using CCK-8 (*n* = 4). (**b**) NO production. Cells were pretreated with each phytochemical (25–100 μM) for 3 h, and further exposed to LPS (100 ng/mL) for 24 h. Produced NO in media was determined using Griess reagent (*n* = 4). Relative mRNA expression of (**c**) iNOS (*NOS2*), COX-2 (*PTGS2*), and (**d**) TNF-α (*TNF*), IL-1β (*IL1B*), IL-6 (*IL6*), and MCP-1 (*CCL2*). Cells were pretreated with catechin (*n* = 4) or taxifolin (*n* = 3), and subsequently stimulated with LPS (100 ng/mL) for 8 h. Significant versus control, *** *p* < 0.001; Significant versus LPS alone-treated group, ^#^ *p* < 0.05, ^##^ *p* < 0.01, ^###^ *p* < 0.001.

A previous study revealed five phytochemicals in QAE such as catechin, isoquercitrin, taxifolin, fraxin, and chlorogenic acid [19]. Five phytochemicals showed no obvious cytotoxicity in BV2 microglial cells at the indicated doses in the current investigation (~100 μM, 24 h) (Figure 7a). Next, we investigated whether phytochemicals of QAE possessed anti-inflammatory activities by measuring NO production in LPS (100 ng/mL, 24 h)-stimulated BV2 microglial cells. Catechin and taxifolin exhibited the most potent inhibitory effect on NO production of the five phytochemicals tested (Figure 7b). Furthermore, catechin and taxifolin significantly suppressed iNOS and COX-2 mRNA expression in response to LPS stimulation (100 ng/mL, 8 h) (Figure 7c). Additionally, catechin and taxifolin also significantly reduced mRNA expression of proinflammatory cytokines (TNF-α, IL-1β, IL-6, and MCP-1) induced by LPS stimulation (100 ng/mL, 8 h) (Figure 7d).

### 3.8. UHPLC-UV-HRMS Analysis of Catechin and Taxifolin in QAE

**Figure 8:**
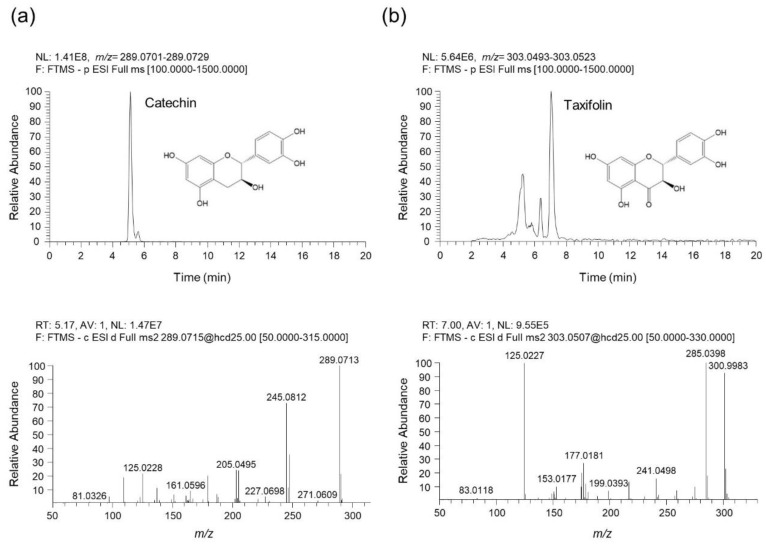
UHPLC-UV-HRMS analysis of catechin and taxifolin in QAE. (**a**) The extracted ion chromatogram (EIC) of catechin identified in QAE (upper chromatogram), and the MS/MS spectrum of catechin (below spectrum). (**b**) The EIC of taxifolin identified in QAE (upper chromatogram), and the MS/MS spectrum of taxifolin (below spectrum). All components were detected in negative ion mode.

To identify the phytochemicals in QAE, an analysis was performed using UHPLC-UV-HRMS. As a result of the analysis, two phenolic components, catechin and taxifolin were identified in QAE. Figure 8 shows the extracted ion chromatograms (EIC), which are chromatograms for the precursor ion *m*/*z* values of each analyte, and MS/MS spectrums. The component identified at retention time (*t*_R_) of 5.07 min presented precursor ions at *m*/*z* 289.0715 [M-H]¯ (Error—0.89 ppm), which was composed of molecular formula C_15_H_14_O_6_ provided by Orbitrap. In addition, the fragment ions of the MS/MS spectrum were *m*/*z* 289.0713, 245.0812, 205.0495, and 125.0228. By comparing these results with previous literature, this component was identified as catechin [19,25]. Taxifolin was identified as follows. Taxifolin detected at 6.99 min showed a precursor ion at *m*/*z* 303.0508 [M-H]¯ (Error—0.92 ppm), which consists of the molecular formula C_15_H_12_O_7_ provided by Orbitrap. The fragment ions shown in the MS/MS spectrum were *m*/*z* 285.0398, 177.0181, and 125.0227. Likewise, it was identified as taxifolin by comparing with the previous literature [19,25,26]. Both catechin and taxifolin identified in QAE were determined by comparing retention time, measured precursor ion, and MS/MS fragments with the values of reference standards, and it was confirmed that the negative ion mode was the optimal condition to for the analysis of both components.

## 4. Discussion

Neuroinflammation has been considered as a significant risk factor for the pathogenesis of neurodegenerative disorders such as Alzheimer’s and Parkinson’s disease [3,6]. Although the anti-hyperuricemia, antibacterial, antiviral activities of QA have been identified, the beneficial effects of QA on neuroinflammation have not been established [15,17,18,19]. Therefore, in the present study, we explored the anti-neuroinflammatory properties of QAE using LPS-stimulated BV2 microglial cells.

Prolonged activation of microglia results in the excessive release of cytokines and neurotoxic molecules, which eventually contribute to neurodegeneration [2]. Thus, controlling microglial activation to reduce the emergence of proinflammatory mediators may be advantageous in managing neurodegenerative disease [6]. First, we studied the effect of QAE on the production of proinflammatory mediators in LPS-stimulated BV2 microglial cells. The results revealed that QAE pretreatment significantly inhibited LPS-induced NO production (Figure 1) and iNOS, COX-2 expressions (Figure 2). Furthermore, it reduced mRNA expression and the release of proinflammatory cytokines including TNF-α, IL-1β, and IL-6 by LPS stimulation (Figure 3). These results suggested that QAE exhibited an anti-inflammatory effect via regulating the production of proinflammatory mediators.

Previous research identified the five major phytochemicals of QAE (taxifolin, chlorogenic acid, fraxin, catechin, isoquercitrin), and isoquercitrin exhibiting the most excellent antiviral efficacy against HSV-1 viral infection [19]. In other reports, taxifolin and catechin isolated from QA inhibited LPS-induced NO production in RAW 264.7 macrophage cells [18]. The inhibitory effect of phytochemicals on LPS-induced NO production in BV2 microglial cells was tested to see if the five phytochemicals of QAE had an anti-neuroinflammatory effect. Among the five constituents, catechin and taxifolin showed the most potent anti-inflammatory effect, reducing LPS-induced NO production (Figure 7), and two phenolic compounds in QAE were identified by UHPLC-UV-HRMS analysis (Figure 8). Furthermore, catechin and taxifolin inhibited LPS-induced mRNA expression of iNOS, COX-2, inflammatory cytokines (TNF-α, IL-1β, IL-6, and MCP-1) (Figure 7b,c). In line with these results, the suppressive effects of catechin and taxifolin on the expression of proinflammatory enzymes in LPS-stimulated BV2 microglial cells were reported previously [27,28]. Based on these findings, the anti-inflammatory activity of QAE is assumed to be connected to the inhibitory efficacy of catechin and taxifolin on the expression of inflammatory mediators.

NF-κB is an essential transcription factor of regulating inflammatory mediators. Signal transduction from TLRs activates IKKα/β, which leads to phosphorylation and degradation of IκB [29]. NF-κB released from IκB translocates to the nucleus and induces the transcription of proinflammatory genes, including iNOS, COX-2, cytokines, and chemokines. In the current study, we revealed that QAE significantly inhibited phosphorylation and degradation of IκB (Figure 4a). Furthermore, immunoblotting results using nuclear protein and immunocytochemistry showed that QAE successfully inhibited the nuclear accumulation of the p65 subunit against LPS stimulation (Figure 4b,c). In addition to nuclear translocation, phosphorylation of p65 on Ser^536^ is essential for transcriptional activation of NF-κB. Evidence showed that phosphorylation of p65 on Ser^536^ by IKKβ enhances NF-κB transcriptional activity, and p65 phosphorylation (Ser^536^) is independent of the IκBα regulation [23,30]. In our study, phosphorylation of p65 (Ser^536^) significantly increased by LPS stimulation, whereas QAE pretreatment effectively reduced the level of p-p65 (Ser^536^) (Figure 4d). We also investigated the effect of QA on the LPS-induced activation of MAPK signaling. QAE pretreatment significantly inhibited LPS-induced phosphorylation of ERK, p38, and JNK (Figure 5). MAPK signaling cascades are involved in regulating cell proliferation, cell survival, and inflammation. It has been known that MAPK activates NF-κB signaling through the degradation of IκB [31]. Moreover, studies using chemical inhibitors showed the abrogation of p38, ERK, and JNK signaling suppressed the production of proinflammatory cytokines such as IL-1β, TNF-α in microglia [32,33]. Thus, the inhibition of the MAPK signaling pathway in microglia is beneficial in resolving inflammation in the CNS. These results suggested that QAE inhibited inflammatory gene expression in the microglial cells by blocking MAPK and NF-κB signaling pathways.

Intracellular ROS acts as key signaling molecules that are implicated in the inflammatory response of microglia [12]. H_2_O_2_ production by LPS-stimulated TLR4 signaling accelerates the activation of NF-κB signaling via phosphorylation and degradation of IκB [11,34]. On the other hand, HO-1, endogenous antioxidant enzyme, and its by-products attenuate TLR4 signaling transduction by inhibiting ROS generation in macrophages [12]. Accordingly, small molecules that activate the Nrf2/HO-1 pathway simultaneously regulate the inflammatory response in BV2 microglial cells [13]. In our study, QAE treatment significantly increased mRNA and the protein expression of HO-1 in a dose-dependent manner, which ameliorated LPS-induced cellular H_2_O_2_ generation (Figure 6).

Nrf2 is a major transcriptional factor regulating the expression of antioxidant genes such as HO-1, GCLC, and SESN2. Nrf2 phosphorylation at Ser^40^ results in translocation to the nucleus. Finally, Nrf2 binds to the antioxidant response element (ARE), and transactivates antioxidant genes, thereby serving cellular redox homeostasis [35]. Apart from the antioxidant response, the anti-inflammatory properties of Nrf2 via the inhibition of the NF-κB pathway and blocking transcription of inflammatory mediators have also been suggested [36]. Representatively, chromatin immunoprecipitation-seq (ChIP-seq) study in macrophages revealed that Nrf2 inhibited the transcription of proinflammatory cytokines by binding to its promoter regions [37]. In our results, QAE successfully activated the Nrf2 signaling pathway by inducing its phosphorylation and nuclear translocation (Figure 6). Taken together, our results suggested that the effect of QAE on Nrf2/HO-1 activation contributes to QA’s anti-inflammatory efficacy.

Overall, our results showed the anti-neuroinflammatory effect of QAE in LPS-stimulated BV2 microglial cells by reducing the expression of proinflammatory enzymes and mediators. These activities were related to NF-κB, MAPK inhibition, and Nrf2/HO-1 activation. Although the present study revealed cellular efficacy and molecular mechanisms of QA on the microglial inflammatory response, efficacy evaluation using neurodegenerative disease experimental animal models needs to be further established for clinical application.

## 5. Conclusions

In the present study, we demonstrated that QAE exhibited anti-inflammatory effects on LPS-stimulated BV2 microglial cells by reducing the expression of proinflammatory enzymes and mediators including iNOS, COX-2, NO, TNF-α, IL-1β, and IL-6. Moreover, we revealed that these inhibitory effects were mediated by inhibiting NF-κB and MAPK signaling pathways. Additionally, we found antioxidative stress efficacy of QAE against LPS-stimulation via the activation of the Nrf2/HO-1 pathway. Thus, QA may be a promising candidate for managing neuroinflammation-mediated neurodegenerative diseases.

## Figures and Tables

**Table 1:** Oligonucleotide sequences used in RT-qPCR experiments.

Gene Symbol	Primer Sequence (Sense, Anti-Sense)	Accession Number	Product Size
*NOS2*	5′-GGCAGCCTGTGAGACCTTTG-3′, 5′-GCATTGGAAGTGAAGCGTTTC-3′	NM_010927.4	72 bp
*PTGS2*	5′-TGAGTACCGCAAACGCTTCTC-3′, 5′-TGGACGAGGTTTTTCCACCAG-3′	NM_011198.4	151 bp
*TNF*	5′-ATGAGCACAGAAAGCATGAT-3′, 5′-TACAGGCTTGTCACTCGAAT-3′	NM_013693.3	276 bp
*IL1B*	5′-ATGGCAACTGTTCCTGAACT-3′, 5′-CAGGACAGGTATAGATTCTT-3′	NM_008361.4	563 bp
*IL6*	5′-TTCCATCCAGTTGCCTTCTT-3′, 5′-ATTTCCACGATTTCCCAGAG-3′	NM_031168.2	170 bp
*CCL2*	5′-TGATCCCAATGAGTAGGCTGG-3′, 5′-ATGTCTGGACCCATTCCTTCT-3′	NM_011333.3	132 bp
*HOMX1*	5′-GGGAATTTATGCCATGTAAA-3′, 5′-AGAACAGCTGCTTTTACAGG-3′	NM_010442.2	294 bp
*GAPDH*	5′-AACGACCCCTTCATTGAC-3′, 5′-TCCACGACATACTCAGCAC-3′	NM_008084.3	191 bp

## Data Availability

All the data are available within the article.
